# Sucrose Monoester Micelles Size Determined by Fluorescence Correlation Spectroscopy (FCS)

**DOI:** 10.1371/journal.pone.0029278

**Published:** 2011-12-28

**Authors:** Susana A. Sanchez, Enrico Gratton, Antonio L. Zanocco, Else Lemp, German Gunther

**Affiliations:** 1 Laboratory for Fluorescence Dynamics, University of California Irvine, Irvine, California, United States of America; 2 Microscopy and Dynamic Imaging Unit, Fundación Carlos-III-CNIC, Madrid, Spain; 3 Laboratorio de Cinética y Fotoquímica, Facultad de Ciencias Químicas y Farmacéuticas, Universidad de Chile, Santiago, Chile; University of Milano-Bicocca, Italy

## Abstract

One of the several uses of sucrose detergents, as well as other micelle forming detergents, is the solubilization of different membrane proteins. Accurate knowledge of the micelle properties, including size and shape, are needed to optimize the surfactant conditions for protein purification and membrane characterization. We synthesized sucrose esters having different numbers of methylene subunits on the substituent to correlate the number of methylene groups with the size of the corresponding micelles. We used Fluorescence Correlation Spectroscopy (FCS) and two photon excitation to determine the translational D of the micelles and calculate their corresponding hydrodynamic radius, R_h_. As a fluorescent probe we used LAURDAN (6-dodecanoyl-2-dimethylaminonaphthalene), a dye highly fluorescent when integrated in the micelle and non-fluorescent in aqueous media. We found a linear correlation between the size of the tail and the hydrodynamic radius of the micelle for the series of detergents measured.

## Introduction

The interest on sugar fatty acid esters (SFAE) started in the mid-1950s, and experienced a renewed attention in the last two decades evidenced by a noteworthy increase of studies in the literature [1]–[6]. These investigations have been motivated by the outstanding surface-active properties (surface tension-reducing capacity, penetrability into lipid bilayers, easiness of dispersion, and remarkable emulsifying power among others) of the SFAE and their environmental friendliness when compared to surfactants derived from petrochemical industry [7], [8]. SFAE are nontoxic and non-allergenic surfactants, readily biodegradable in aqueous environments [8], [9]. Additionally, the raw materials involved in their synthesis (fatty acids or their derivatives and sucrose) are low cost, simple, easily accessible and renewable [10]–[13]. SFAE have a broad range of applications going from technological fields such as cosmetic and health care [2], [14] to food additives [2].

These non-ionic carbohydrate-based surfactants can contain among others sucrose [3] as hydrophilic head. The hydrophobic tail of these compounds corresponds to a hydrocarbon chain (of different length and degree of insaturations) substituting one or more specific hydroxylic groups on the sucrose moiety. The substitution and purity of these compounds usually depends on the synthetic method and separation techniques employed.

Micelle forming surfactants are used frequently in biochemical studies to solubilize integral membrane proteins, SFAE are not an exception. Derivatives of stearic acid had been employed for the extraction of cytochrome and lysozyme [15]–[17]. Size and shape of micelles are determinant in the packing of the surfactant tails, and consequently in the conformation of proteins incorporated in their hydrophobic core and in the protein-surfactant interactions [18]. The micelle properties determine the optimal surfactant conditions for extraction, purification, structural and functional characterization of membrane proteins.

Surfactants are also used to obtain supramolecular mesoporous materials utilized as templates for other applications [19]. The characteristic of the formed mesostructure will depend on the length of the hydrocarbon chain and the surfactant tail, according the ‘liquid crystal templating mechanism” [19]. The mesophase formation is directly related with the packing of hydrocarbon tails, property related with the surfactant packing parameter, (V/a_0_l), where V is overall volume of the surfactant, a_o_ is the effective head group area, and l is the surfactant chain length [19]. The dimensional values involved in the mesophase formation can be achieved by knowing the size and the physicochemical properties of the micelles formed by the surfactants.

The properties of the micelles (aggregation number, diffusion coefficient and size) can be determined by using diffusional NMR methods, pulsed field gradient spin-echo NMR (PFGSE–NMR) [6], dynamic light scattering (DLS) [20], [21], photon correlation spectroscopy (PCS), small angle neutron scattering (SANS) [22] and fluorescence based methods such as steady-state fluorescence quenching (SSFQ) time-resolved fluorescence quenching (TRFQ) [23] and Fluorescence Correlation Spectroscopy (FCS) [22], [24]–[27].

Most of the non-fluorescence based methods are restricted to small values of the aggregation number (thermodynamic methods, NMR) or the measurements depend on the micelle shape and inter-micellar interactions (scattering methods) therefore, in order to extract the value of the aggregation number, the results must be extrapolated to low concentration, close to the cmc. Instead, the fluorescence based methods, allow determinations of micelle aggregation numbers under the actual experimental conditions without being affected neither by intermicellar interactions nor by the micelle shape [28].

On the other side, one of the disadvantages reported for the fluorescence based methods is the overestimation of N_agg_ when compared with the ones determined by NMR procedures, probably, due to the incorporation of fluorescent probes (usually big in size). This disadvantage becomes more dramatic when the fluorescent probe used has also surfactant properties, in such a case, even at low concentration of the probe, the microaggregate formed by a mixture of surfactants (the fluorescent probe and the surfactant under study) would have significantly different physicochemical properties when compared with micelles of the single surfactant [29], [30]. However, different fluorescent dyes have been used for the determination of aggregation number in similar systems and the results showed to be consistent and independent of the dye used, for instance: the translational diffusion coefficients, hydrodynamic volumes and aggregation numbers for micelles of several surfactants (deoxycholate, CTAB, SDS, Tween 80 and Triton X-100) reported by Hink et al., using FCS and octadecyl rhodamine B chloride (ODRB) or NBD derivative were similar and in the same range than the one recently determined by FCS for Tween 20 micelles loaded with 9,10-bis (phenylethynyl) anthracene (BPEA) [27]. These results are in good agreement with the values reported in the literature using pyrene in SSFQ and TRFQ studies [23]. Gapinski et al. [22], compared the advantages of different techniques (SANS, PCS and FCS) to evaluate the properties of the hexaethylene glycol monododecyl ether (C_12_E_6_)/water system (rod-like micelles with elliptical cross section) in the isotropic phase, and they reported the valuable contribution of FCS measurements to the study of micellar behavior. Meng and Russel [21] described a theory to predict the N_agg_ and the radius of spherical micelles formed by telechelic associative polymers using the molecular structure and a few parameters like the surface tension of hydrophilic and hydrophobic blocks of the polymer and of the solvent. Although the values predicted by this theory agreed with the measured data reported in the literature, the method is limited by the availability of parameters for polymers with other hydrophobic blocks.

Fluorescence correlation spectroscopy (FCS) uses the temporal fluctuations of fluorescence to determine several physical or chemical parameters of the particle producing the fluctuations. Translational diffusion coefficients, flow rates, chemical kinetic rate constants, molecular weights and aggregation have been determined using FCS [28].

In our previous studies, physico-chemical information (critical micelar concentration, and several properties such as micropolarity, microfluidity, shape and aggregation number) of the micellar aggregates formed by pure 6-O sucrose esters has been reported [3], [6], [31]. In this work we report the translational diffusion coefficients of a complete series of sucrose esters as a function of chain length using FCS and Laurdan (6-dodecanoyl-2-dimethylaminonaphthalene). We discuss the data and compare them with the data obtained employing diffusional NMR methods [6]. Our results show a linear relationship between the size of the micelle and the tail length for the series of sucrose surfactants used and confirms the strength of FCS in the study of micro-heterogeneous systems.

## Results and Discussion

Sucrose esters with different hydrocarbon tail length have particular physicochemical properties such as membrane solubilization capacity, or partition coefficient [32], [33], relevant for applications in protein extraction [15]–[17] and mesomorphic surfaces formation studies [19]. One of this properties is the size of the micelle which is determined in some extent by the number of methylene units in the hydrophobic tail. We have synthesized a series of sucrose esters with different tail size and determined the effect of the chain length on the size of the micelle formed by the detergent using Fluorescence Correlation Spectroscopy and two-photon excitation.

**Figure 1:**
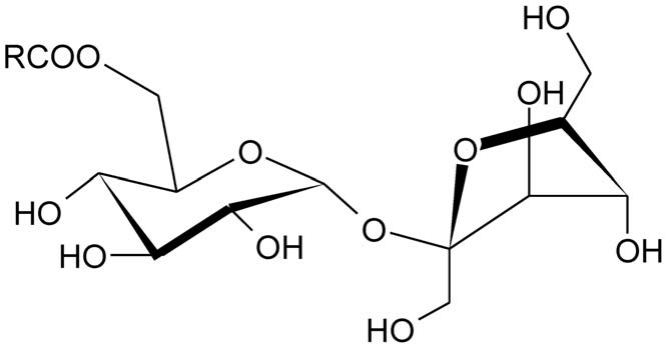
Chemical structure of studied sucrose monoesters, R = C_7_H_15_ Octyl acid derivative (MOS), R = C_9_H_19_ Capric acid derivative (MCS), R = C_11_H_23_ Lauric acid derivative (MLS), R = C_13_H_27_ Myristic acid derivative (MMS), R = C_15_H_31_ Palmitic acid derivative (MPS), R = C_17_H_35_ Stearic acid derivative (MSS).

Fig. 1 shows the chemical structure of the different sucrose esters used in these experiments, the nonionic head group corresponds to a sucrose moiety (highly substituted with hydroxyl groups) and the hydrophobic tail is a hydrocarbon chain of increasing length in the series. The series go from 10–18 methylene units. The fluorescent probe employed, Laurdan, has the advantage of having a high partition coefficient into the organic phase, (i.e. low water solubility), and so observed fluorescence arises exclusively from micelles without the interference of the free dye [34]. The results were not influenced by sucrose ester concentration indicating that micelles maintain their aggregation number and shape in the working concentration range. For most amphiphile solutions morphological transformations take place over certain concentration range, and the behavior observed when the sample is constituted by more than one surfactant deserves special attention. The changes in the interactions between components affect the free energy of micellization and hence, aggregation parameters like N_agg_ and size are very different from the ones predicted by an ideal behavior (even at low compositions). Also changes in the mixed micelle composition have been observed as a function of total surfactant concentration [29], [30]. Size and shape of the micelles can also be modified when additives are inserted in the interphase, for example, the incorporation of catechol on CTAB micelles changed head interactions and packing parameters, inducing an increase in the size of the aggregates and the sphere-to-rod morphological change [35].

**Figure 2:**
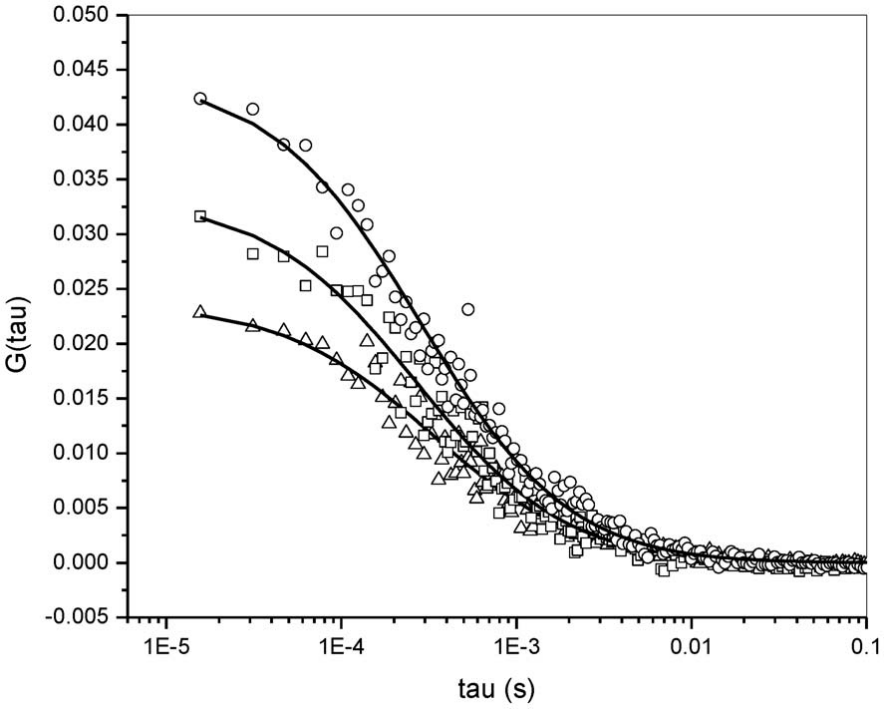
Plot showing the autocorrelation functions obtained from the samples of different sucrose esters loaded with Laurdan 20 nM. The symbols show the experimental autocorrelation (○ MMLS □ MMCS ◊ MPS) and the solid line the fitting of the data using a Gaussian-Lorentzian model for diffusion. The diffusion coefficient obtained were 38.9, 39.9 and 34.3 µm^2^/s for MMLS, MMCS and MPC respectively.

**Figure 3:**
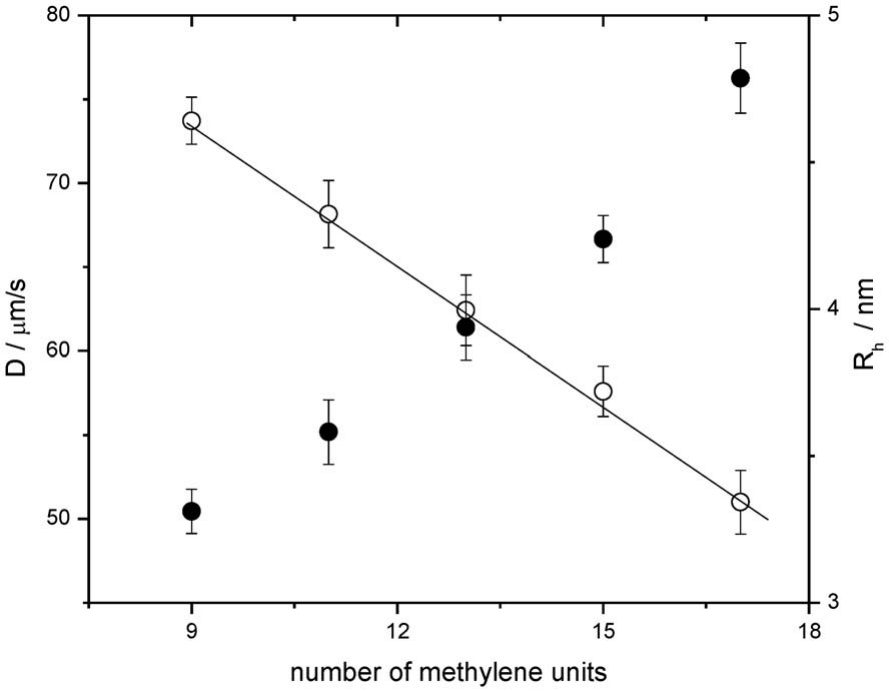
Plot showing the dependence of translational diffusion coefficients, D ○, and the hydrodynamic radius, R_h_ •, for micelles of alkyl sucrose esters against the length of the alkyl substituent.

Fig. 2 shows an example of the experimental autocorrelation curves obtained from micelles samples of different sucrose esters loaded with Laurdan 20 nM. In those experiments the surfactant concentration for the different sucrose esters was kept over their corresponding cmc [31]. The diffusion coefficient (D) for the different samples was obtained by fitting of the experimental autocorrelation curves to a Gaussian-Lorentzian model for diffusion (see methods) and they are reported in Table 1. The values for the D for the series go from 51.00±0.12 (MSS) to 73.7±0.08 (MCS) µm^2^/s. Our data are similar to the ones reported for MPS and MCS by Molinier et Al. using NMR procedure (see Table 1) [6]. For the set of surfactants studied, the dependence of D with the number of carbon units in the hydrophobic tail (methylene units plus carbonyl) shows a linear correlation (Fig. 3) and consequently there is a linear dependency of the hydrodynamic radius with the length of the alkyl chain (Fig. 3). Linear fit of the R_h_ data in Fig. 3 gives a slope equal to 181 pm (in the range of a C-C bond (154 pm)) and an intercept of 1.44 nm. This last value corresponds to the length of the region occupied by the sucrose moiety plus the structured water, and it is 20–40% overestimated when compared with the reported hydrodynamic diameter for sucrose in water (1.00–1.12 nm) [36]. Our result showing the micelle hydrodynamic radius changing linearly with the length of alkyl chain, can be interpreted as follows: when the chain increases (longer sucrose ester derivative), a new volume is available in the structure and it is occupied by an additional surfactant molecule (increasing the aggregation number and the surface area of micelles). Thus, additional surfactant molecules will fill up the increased surface area maintaining the density of the micelle.

**Table 1:** Experimental diffusion coefficients and calculated values hydrodynamic radius and aggregation numbers for micelles of sucrose 6-O monoesters with different length of alkyl chain.

	MSS	MPS	MMS	MLS	MCS
D/(µm^2^ s^−1^)	51.00±0.12	57.60±0.08	62.0±0.11	68.2±0.11	73.7±0.08
D[6][6]/(µm^2^ s^−1^) [6]		57			82
R_h_/(nm)	4.79	4.24	3.94	3.58	3.31
A/(Å^2^ molec^−1^) [31]		105.8	66.2	45.6	50.2
N_agg_ (a)		21.4	29.5	35.3	27.4
N_agg_ (b)	178	139	120	99	85
N_agg_ [31]		110	90	80	
N_agg_ [1]		160	122	96	76

When protein-detergent complexes are to be separated based on the molecular size of the protein, smaller micelles are more easily removed and hence are usually desirable. The parameter evaluated for downstream removal is the micelle molecular weight, directly related with the size of micelle. The micelle molecular weight is simply the product of the aggregation number (N_agg_) and the monomer molecular weight. The determination of the aggregation number N_agg_ (the average number of detergent molecules in a micellar unit) is therefore of great significance.

A relatively simple method for calculating mean aggregation numbers of surfactant solutions uing fluorescent procedures, requires a hydrophobic probe and a quencher greatly partitioned in the micelle [37]. To calculate N_agg_ from diffusional parameters, Hink et al. [28] used some geometrical considerations (assuming spherical micelles) and Eq 1
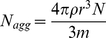
(1)where ρ is the mean density of the micelle (g cm^−3^), m is the molecular mass of a surfactant molecule (g mol^−1^) and N is Avogadro's number. However, this approach yields poor results when compared with traditional static or dynamic fluorescence determinations [37].

Another equivalent approach (method A) to achieve N_agg_ from diffusional data, avoiding the use of density, is the use of the cross-sectional area per molecule of each ester determined by surface tension measurements [31] and the micellar spherical volume from the diffusional data. Table 1 presents the N_agg_ calculated using method A. For comparison we have included in the same Table 1 N_agg_ determined by time resolved and static fluorescence quenching methods [31] and by X-ray scattering [1]. The values determined using the diffusion parameters are, on the contrary of Hink determinations, between two and four times smaller than the ones previously reported. The origin of this difference is probably in the use of cross-sectional area per molecule obtained from monolayers.

**Figure 4:**
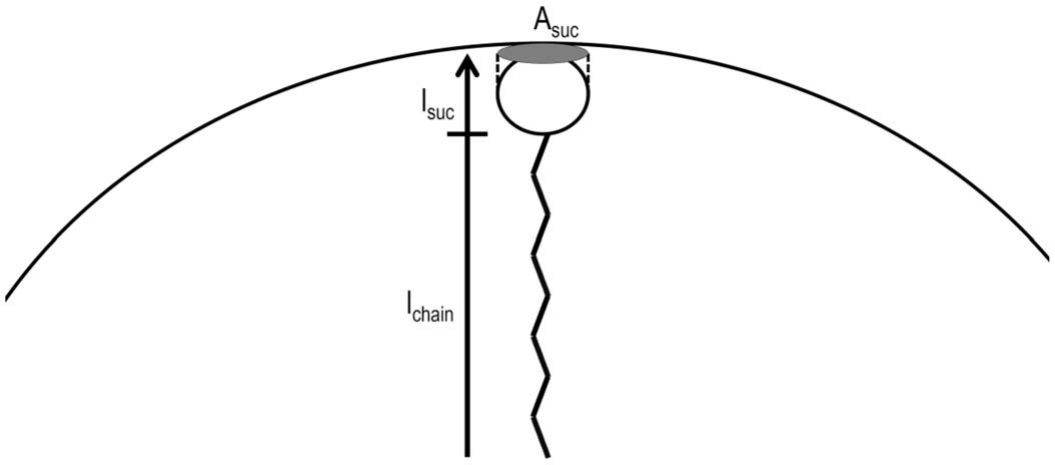
Scheme showing the geometrical considerations to obtain aggregation numbers of micelles from FCS data. Open circle represent the sucrose moiety and the zig-zag tail a carbon tail.

We propose a self-consistent calculation of N_agg_ (method B) based on the geometrical assumptions shown in Fig. 4 and using the values obtained by FCS measurements. The micelle hydrodynamic radius, R_h_, corresponds to the contribution of two components: the length of the alkyl chain (l_chain_) and the sucrose hydrodynamic diameter (l_suc_), obtained from intercept of plot shown in Fig. 3:
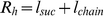
(2)The micelar surface occupied by one polar head can be calculated as the circular projection of sucrose head (supposed spherical). Under these considerations:
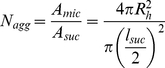
(3)Replacing Eq 2 on Eq 3 we obtain the aggregation number from:
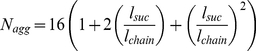
(4)The N_agg_ determined for the series of detergents using our method (method B in Table 1) are in good agreement with the data determined using other experimental methods [1], [31], however, the values for l_chain_ obtained using eq 3 are 30% larger than the values obtained with Tanford equation (l_chain_ = (0.154+0.1256 n) nm [38]), relationship normally used for l_chain_ calculation in micellar systems. It will be interesting to test our method with other micellar systems, in fact this method may be also an improved method to measure l_chain_ for specific surfactants, then, careful comparative studies will be needed to clarify this point.

Finally, diffusion coefficient values determined at several ester concentrations, for all the studied compounds show no dependence on ester concentration, indicating that micelles do not aggregate and remained as individual entities in the studied range (1–20 mM). At this point it must be stated that the relation of micelar volume with the number of methylene units of hydrophobic tail is polynomial, involving the size of the polar head, so the experimental observation of an apparently linear dependence of diffusion coefficients, probably has its origin in the range of lengths involved in the measurement.

In previous reports FCS technique has been used in combination with other measurements as a useful tool for estimating micelar size and shape, now we can state that when measurements are performed for a series of surfactants, self-consistent results related with micellar size and N_agg_ can be achieved with FCS determinations alone. The data analysis we propose (method B) involves a simple and reliable procedure which yields results fully comparable with the ones obtained by other procedures.

## Materials and Methods

### Chemicals

Sucrose monoesters β-d-fructofuranosyl-6-O-capryl-α-d-glucopyranoside (Mono capryl sucrose, C10), β-d-fructofuranosyl-6-O-lauryl-α-d-glucopyranoside (Mono lauryl sucrose, C12), β-d-fructofuranosyl-6-O-miristyl-α-d-glucopyranoside (Mono miristyl sucrose, C14), β-d-fructofuranosyl-6-O-palmityl-α-d-glucopyranoside (Mono palmityl sucrose, C16) and β-d-fructofuranosyl-6-O-stearyl-α-d-glucopyranoside (Mono stearyl sucrose, C18), were synthesized under Mitsunobu conditions, according the procedure reported by Molinier et al. [13], by using sucrose (Merck) and the corresponding carboxylic acid (from Sigma). The reaction yields a relatively complex mixture of two monoesters (6-O and 6-O'), one diester (6-O, 6-O') and two monoesters of anhydrosucrose. Semipreparative chromatography on silica and amino modified C18 silica column were employed to isolate the pure 6-O monoesters. Briefly, the reaction mixture was solubilized in chloroform and eluted from a semipreparative silica gel column by using chloroform:methanol mixtures as mobile phase (the proportions were dependent on the specific sucrose monoester isolated). The final purification of the fraction containing monoesters was made in the C18 amino column with acetonitrile:water mixtures as a mobile phase. Thin layer chromatography (using the first mobile phase and staining with a butanolic solution of urea-orthophosphoric acid) showed mainly one compound in the purified sample. The NMR spectra, obtained in a Bruker ADX 300 spectrometer, in DMSO_d-6_ containing 5% of CH_3_OD to avoid micellization, are in good agreement with previously reported spectra for several monoesters [10], [39].

6-Dodecanoyl-2-dimethylaminonaphthalene (Laurdan) from Invitrogen was used as received. Fluorescein standard was obtained from Sigma. All solvents employed were from Merck and HPLC quality.

### Sample preparation

Surfactant solutions were prepared by weighting the appropriate amount of the corresponding sucrose ester, dissolving in water to final concentration around 0.1–20 mmol L^−1^ and sonicating them during at least one hour. After sonication, Laurdan was added from an Stock solution (1.0 µmol L^−1^ in DMSO) to a final concentration 10–20 nM and stirred gently for 1 hour at room temperature. With the concentrations of surfactant and dye employed, low mean numbers of occupation are assured.

### Fluorescence Correlation Spectroscopy

FCS measurements were performed in a two-photon fluorescence microscope designed at the Laboratory for Fluorescence Dynamics [40], [41]. A mode-locked titanium-sapphire laser (Mira 900, Coherent, Palo Alto, CA) pumped by a frequency-doubled Nd:vanadate laser (Verdi, Coherent) and set to 780 nm was used as the two-photon excitation light source. For all measurements, the average power used at the sample ranged from 10–18 mW. A miniature photomultiplier (R5600-P, Hamamatsu, Bridgewater, NJ) was used for light detection in the photon counting mode and counts were acquired using a ISS card (ISS, Inc., Champaign, IL). Data acquisition was performed using the SimFCS program (Laboratory for Fluorescence Dynamics, UCI, CA). A UPlan Apo/IR 60X/1,20 (Olympus, Japan) water objective was used for all the measurements.

Experimental autocorrelation functions were fitted assuming a Gaussian-Lorentzian intensity profile, as described in a previous work which contains the explicit formulas for the point spread function and the definition of the beam waist used [42]. The beam waist of the Gaussian-Lorentzian function depends on the instrument setup and must be calibrated each time the system is aligned. For this purpose a substance with a known concentration and diffusion coefficient (D) was used to calibrate the excitation volume. In this work, fluorescein (20 nM in Tris, pH>9), with a diffusion constant of 300 µm^2^/s was used as a standard [43]. The recovered beam waist value (W_0_ = 0.35 µm) was used to perform the analysis with each set of data.

From the fitted D values it is possible to calculate the hydrodynamic radius (R_h_) of the molecule responsible of the detected intensity fluctuations using the Stokes-Einstein relation:
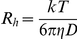
Where, k is the Boltzmann constant, T the temperature, η the media viscosity (considering the low concentrations employed, solutions viscosity was considered to be equal to the one of water) and finally, D corresponds to the diffusion coefficient of the species in motion.
