# Correction: Sucrose Monoester Micelles Size Determined by Fluorescence Correlation Spectroscopy (FCS)

**DOI:** 10.1371/journal.pone.0125123

**Published:** 2015-04-17

**Authors:** Susana A. Sanchez, Enrico Gratton, Antonio L. Zanocco, Else Lemp, German Gunther

The following information is missing from the Funding section: SAS thanks the Fondo Nacional de Desarrollo Científico y Tecnológico (FONDECYT) 1140454, http://www.conicyt.cl/fondecyt/.
